# Bovine pericardial patch in an eggroll fashion and teflon felt microvascular decompression for trigeminal neuralgia and hemifacial spasm: A retrospective study

**DOI:** 10.1097/MD.0000000000047074

**Published:** 2026-01-16

**Authors:** Min Tang, Seidu A. Richard, Rong Liu, Zhigang Lan, Heng Zhang, Ding Lei

**Affiliations:** a Department of Neurosurgery, West China Hospital, Sichuan University, Chengdu, Sichuan, China; b Institute of Neuroscience, Third Affiliated Hospital, Zhengzhou University, Zhengzhou, China; c Department of Biochemistry and Forensic Sciences, School of Chemical and Biochemical Sciences, C. K. Tedam University of Technology and Applied Sciences (CKT-UTAS), Navrongo, Ghana.

**Keywords:** BPP, Eggroll, HFS, MVD, Teflon felt, TGN

## Abstract

Teflon felt (TFF) is widely adopted for microvascular decompression surgeries (MVDS) for hyperactivity syndrome involving the trigeminal neuralgia (TGN) and hemifacial spasm (HFS) while bovine pericardial patch (BPP) used for dural graft and not MVDS. We speculated that BPP roll around the nerve like an eggroll and fixed with a closing suture may solve the complications associated with TFF dependent MVDS and reduce hospital cost. We conducted a comparative retrospective study on all MVDS for TGN and HFS performed at the department of Neurosurgery, West China Hospital from January 1, 2015, to January 1, 2022. Data like clinical and imaging characteristics, operative procedures, postoperative complications, recurrence rate were recorded and compared between patients who underwent TFF and BPP MVDS. We observed a significant statistical difference (*P* = .0135) in recurrence and granuloma formation between the TFF and BPP MVDS. The time of recurrence and the annual rate of control of symptomatology were almost same in both groups until 30 months onwards where cases of recurrences were high in the TFF MVDS (4.2% vs 0%; *P* = .0135, 95% CI: 1.8–6.5%). Also, total cost was relatively lower in BPP MVDS compare to the TFF MVDS (*P* = .00135). BPP roll around the nerve like an eggroll and fixed with a closing suture could be a good alternative for TFF dependent MVDS. Notably, the BPP MVDS had no granuloma formation and no recurrence of symptomatology.

## 1. Introduction

Hyperactivity syndrome involving the trigeminal neuralgia (TGN) and hemifacial spasm (HFS) are well-known phenomena associate with cranial nerves which often manifests as severe disabling facial pain as well as spasm.^[1–5]^ Microvascular decompression surgeries (MVDS) for TGN and HFS offers a reasonably low-risk prospect to treat these cranial nerve hyperactivity syndromes.^[2,6]^ However, the implantation of a prosthesis during MVDS is still a matter of debate with some neurosurgeons having contradiction to the placement of any material in contact with the nerve root.^[2,6]^

The widely used prosthesis during MVDS are Ivaron sponge or Teflon felt (TFF).^[1,7]^ These prostheses are occasionally associated with granuloma formation around the implants, as well as adhesive inflammation and arachnoiditis which triggers recurrence although they are safe in most cases.^[1,8–15]^ Additionally, separation of the prosthesis and the cranial nerve is often challenging and associated with high risk of damages to the nerve if a second operation is required in cases of recurrent symptomology or failed MVDS.^[1,9,11,16–19]^

Furthermore, although TFF is widely adopted for MVDS, it is also associated with insufficient decompression in addition to the site back above. Bovine pericardial patch (BPP) has been widely used for dural graft^[20–22]^ and vascular reconstructions,^[23]^ but not MVD surgeries for TGN and HFS. We speculated that BPP roll around the nerve like an eggroll and fixed with a closing suture may solve the complications and provide a good alternative for TFF dependent MVDS. The prime focus was complications and recurrence between patients treated with TFF MVDS and BPP MVDS. Also, we compared the average length of hospital stay (LOS) and hospital cost between the BPP MVDS and the TFF MVDS.

## 2. Materials and methods

We conducted a comparative retrospective study on all MVDS for TGN and HFS performed at the department of Neurosurgery, West China Hospital from January 1, 2015, to January 1, 2022. This study was approved by the Ethic Committee of West China Hospital; Sichuan University. The patients and their relatives fully consented to the use of their information after dually informing them about our intention to involve them in a study. All the patients signed written informed consents for publication. Patients who underwent TFF MVDS were compared to patients who underwent BPP MVDS. Data like clinical and imaging characteristics, operative procedures, postoperative complications, recurrence rate, LOS and hospital cost were recorded and compared for all the patients who underwent these procedures. All methods were performed in accordance with the relevant guidelines or protocols and regulations. Sample size estimation was performed using GPower 3.1. Based on historical recurrence rates (TFF MVDS: 6%, BPP MVDS: 1%), a power analysis (α = 0.05, β = 0.2, effect size = 0.15) indicated a minimum requirement of 354 patients per group. The final cohort (BPP MVDS: 368, TFF MVDS: 430) exceeded this threshold.

### 2.1. Sample size calculation

#### 2.1.1. Statistical parameters

Type I error (α) = 0.05, Type II error (β) = 0.2 (80% power); Historical recurrence rates: TFF MVDS = 6%, BPP MVDS = 1% (effect size = 5%); and Minimum required sample size: 354 patients per group (G*Power 3.1, χ² test).

#### 2.1.2. Final cohort

TFF group: 430 patients; BPP group: 368 patients; Rationale: Final sample size exceeded the calculated minimum threshold.

### 2.2. Clinical characteristic and Imaging

The clinical characteristic such as sex, age at onset of symptoms, age at surgery and preoperative duration of symptoms in years as well as prior drug treatment (PDT) of all the patients included in the study were obtained and documented. Also, all patients included in this study underwent brain magnetic resonance imaging (MRI) and computed tomography (CT) scans to exclude all structural pathologies.

### 2.3. Surgical procedure

**Figure 1.**
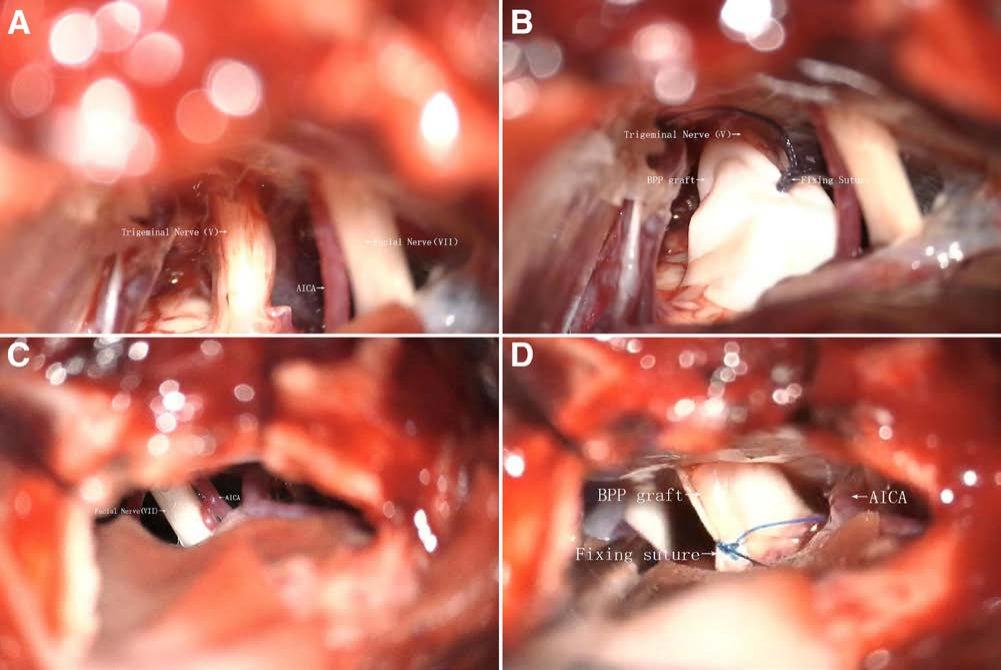
Are intraoperative in images of patients who underwent BPP MVDS for TGN and HFS. (A) Is an intraoperative image showing the trigeminal nerve (V), facial nerve (VII) and the anterior inferior cerebellar artery (AICA). (B) Is an intraoperative image showing the trigeminal nerve (V), with BPP in eggroll fashion and fixed with a suture. (C) Is an intraoperative image showing the facial nerve (VII) and the anterior inferior cerebellar artery (AICA). (D) Is an intraoperative image showing the facial nerve (VII), with BPP in eggroll fashion and fixed with a suture. BPP = bovine pericardial patch, HFS = hemifacial spasm, MVDS = microvascular decompression surgery, TGN = trigeminal neuralgia.

All MVDS (TFF and BPP) were conducted via a standard retrosigmoid approach and techniques were performed by a neurosurgeon with over 15 years of experience in the performing MVDS. After releasing cerebrospinal fluid (CSF) under the microscope, the cerebellar hemispheres were retracted. The arachnoid plane between the responsible vessels and the target nerves (trigeminal or facial) were opened and the cisternal segments of the nerves were exposed. The entire lengths of the nerve roots; from the brainstems exit zones to the entrances of the Meckel caves or internal auditory canals were dissected. In the BPP MVDS, after the offending vessels were separated, the nerves were loosely wrapped by a BPP like an eggroll and the ends of patch fixed together with a polyglycolic acid or prolene (polypropylene) absorbable closing sutures (Fig. 1A–D). The BPP was loosely wrapped around the nerve, maintaining a 2 to 3 mm gap (confirmed intraoperatively with a spacer tool) to avoid compression. Sutures (6–0 Prolene) were secured under 0.5 to 1.0 N tension, measured with a spring scale (Fig. 1B). The size of the BPP was often as per the length and width of the nerve involved after exposure. The same BPP was used as patch and dura repair during operations in all the patients in the BPP MVDS. In the TFF MVDS, we used polytetrafluoroethylene material which is herein referred to as TTF for the procedures. Also, in the TFF MVDS, the TFFs were interposed between the offending vessels and nerves to maintain separations. The Teflon mini (TFM) plates separated the offending vessels from the nerve as per our institution protocols. There were no signs of blood impregnation of the Teflon felt in all the patients. Furthermore, in all patients, electromyogram (EMG) as well as auditory brainstem responses (ABRs) were utilized to monitor the cranial nerves. Recurrent cases were reoperated and samples of tissues that triggered recurrences were evaluated via histopathology.

### 2.4. Outcome assessments

Successes of the operations, or excellent outcomes, were defined as the reduction in symptoms such as lancinating facial pain or spasm at least 95% compared with the level preoperatively, as assessed by the patients, without pain and medications for tics. Also, routine history and examination was used to establish recurrence of symptomatology during follow-ups. Additionally, relevant diagnostic modalities and gradings/scores were used to assess and/or establish complications in all the MVDS as per our hospital protocols. MRI and CT scan was used to evaluate cases with recurrences as well as establish initial signs of granuloma formation. However, histopathology was used to establish definitive diagnosis of granulomas.

Also, MRI and CT scan was used to establish brain stem infarction, cerebellar edema, hydrocephalus, infection and implant displacement between the 2 groups. Facial paresis and hearing loss were established with EMG as well as ABRs and physical examination while CSF leakage was established by physical examination. Intracranial infection was established by high fever >37°C as well as CSF cultures and sensitivity. Routine follow-up visits were scheduled as per our hospital protocols. Follow-ups of all the patients ended on December 31, 2022. The primary endpoint was complications and recurrence. Treatment success required >95% symptom reduction confirmed by both patient self-report and objective scales: Barrow Neurological Institute Pain Scale (BNI-PS) for TGN and House–Brackmann Grading System for HFS.

### 2.5. Statistical analysis

Data was entered and preserved for analysis in the SPSS software version 23.0 (IBM Corp., Armonk). The results of Fisher exact test are reported for two-by-two tables; the Mann–Whitney test was used to assess the significance of ordinal variables, and the unpaired *t*-test or analysis of variance was used for continuous variables. Kaplan–Meier curves were calculated with the Turnbull modification for interval-censored success data. We calculated the postoperative rate of recurrence using life table analysis, with confidence intervals from Gehan variance estimate. All statistical tests were two-tailed. *P* < .05 was considered statistically significant. Multivariable Cox regression models adjusted for age, sex, symptom duration, and vessel type (artery vs vein) were used to analyze recurrence and complications.

## 3. Results

### 3.1. Patients diagnosis and type of surgery

In all, a total of 798 patients who underwent TGN and HFS surgeries were included in the study and out of them, 644 had TGN surgeries while 154 had HFS surgeries (Table 1). Also, out of the 798 patients, 368 underwent BPP MVDS while 430 underwent TFF MVDS. Out of the 368 patients who underwent BPP MVDS, 294 patients had TGN while 74 patients had HFS. Furthermore, out of the 430 patients who underwent TFF MVDS, 350 patients had TGN while 80 patients had HFS.

**Table 1:** Patients’ characteristics between 2 groups.

Parameters	BPP MVD (N = 368)		TFF MVD (N = 430)		*P*-value
Patients’ diagnosis & type of surgery	Trigeminal neuralgia (294)	Hemifacial spasm (74)	Trigeminal neuralgia (350)	Hemifacial spasm (80)	
Sex					
Male	98	24	110	30	.9261*
Female	196	50	240	50	
Age at onset of symptoms	48 ± 2	49 ± 3	50 ± 2	48 ± 2	.8254**
Age at surgery	56 ± 3	54 ± 4	53 ± 4	57 ± 2	.3567***
Preoperative duration of symptoms (yr)	4 ± 2	5 ± 1	5 ± 2	6 ± 1	.2453
Side of operation					
Left	95	23	115	19	.1258*
Right	190	49	229	57	
Bilateral	9	2	6	4	
Prior drug treatment					
Yes	292	70	349	78	.8144*
No	2	4	1	2	
Responsible vessels					
Superior cerebellar artery	219	3	310	3	.3727**
Anterior inferior cerebellar artery	20	65	15	63	
Posterior inferior cerebellar artery	5	2	1	2	
Labyrinthine artery	3	5	2	1	
Unspecified small artery	3	1	4	2	
Vein only	6	1	5	19	
Vein and artery	34	14	30	10	
Unspecified small artery or vein only	3	4	4	2	

There are no significant statistical difference in sex, prior drug treatment (PDT), preoperative duration of symptoms, age at onset of symptoms, age at surgery as well as the side of operation in both TGN and HFS patients well as in both the BPP MVD and the TFF MVD respectively.

BPP = bovine pericardial patch, HFS = hemifacial spasm, MVD = microvascular decompression, TFF = Teflen felt, TGN = trigeminal neuralgia.

Statistical analysis was done as follows;

* Fisher exact test,

** Mann–Whitney test,

*** Unpaired *t*-test test. Unspecified small arteries and veins denotes vessels < 1 mm diameter without identifiable origin from named cerebellar/basilar artery branches.

### 3.2. Clinical characteristic and imaging outcome

Out of the 798 patients (Table 1), 262 patients were males while 536 patients were females. Out of the 368 patients included in the BPP MVDS, 122 patients were males while 246 patients were female. Furthermore, out of the 368 patients included in the BPP MVDS, 98 males had TGN while 24 males had HFS. Also, out of the 368 patients included in the BPP MVDS, 196 females had TGN while 50 females had HFS.

In the TFF MVDS, out of the 430 patients, 140 patients were males while 290 patients were female. Additionally, out of the 430 patients included in the TFF MVDS 110 males had TGN while 30 males had HFS. Furthermore, out of the 430 patients included in the TFF MVDS 240 females had TGN while 50 females had HFS.

In the BPP MVDS (Table 1), the average age at onset of symptoms was 48 ± 2 years in TGN patients and 49 ± 3 years in the HFS patients. Also, in the TFF MVDS, the average age at onset of symptoms was 50 ± 2 years in TGN patients and 48 ± 2 years in the HFS patients. The average age at surgery was 56 ± 3 years in TGN patients and 54 ± 4 years in the HFS patients in the BPP MVDS. Moreover, the average age at surgery was 53 ± 4 years in TGN patients and 57 ± 2 years in the HFS patients in the TFF MVDS.

In the BPP MVDS, the average preoperative duration of symptoms was 4 ± 2 years in TGN patients and 5 ± 1 years in the HFS patients. Also, in the TFF MVDS, the average preoperative duration of symptoms was 5 ± 2 years in TGN patients and 6 ± 1 years in the HFS patients. Out of the 798 patients, 789 patients had PDT before surgeries while 9 patients denied any PDT before surgeries. The distribution in terms of BPP and TFF grouping as well as TGN and HFS are as shown in (Table 1). All patients included in this study underwent brain both MRI and CT prior to surgeries.

### 3.3. Surgical outcome

All the patients underwent surgeries to relieve their symptomatology. In the BPP MVDS, out of the 368 patients who underwent the operations, 95 patients had surgeries on their left sides in the TGN patients while 23 patients had surgeries on their left sides in HFS patients. Also, 190 patients had surgeries on their right sides in the TGN patients while 49 patients had surgeries on their right sides in HFS patients. Furthermore, 9 patients had surgeries on both sides in the TGN patients while 2 patients had surgeries on both sides in HFS patients (Table 1).

In the TFF MVDS, out of the 430 patients who underwent the operations, 115 patients had surgeries on their left sides in the TGN patients while 19 patients had surgeries on their left sides in HFS patients. Also, 229 patients had surgeries on their right sides in the TGN patients while 57 patients had surgeries on their right sides in HFS patients. Furthermore, 6 patients had surgeries on both sides in the TGN patients while 4 patients had surgeries on both sides in HFS patients (Table 1).

In the BPP MVDS, out of the 368 patients who underwent the operations (Table 1), superior cerebellar artery (SCA) was responsible for TGN in 219 patients and 3 patients HFS. Anterior inferior cerebellar artery (AICA) was responsible for TGN in 20 patients and 65 patients in HFS. Posterior inferior cerebellar artery (PICA) was responsible for TGN in 5 patients and 2 patients in HFS. Labyrinthine artery (LA) was responsible TGN in 3 patients and 5 patients in HFS.

Furthermore, out of the 368 patients, unspecified small arteries were responsible TGN in 3 patients and 1 patient in HFS. Vein only was responsible TGN in 6 patients and 4 patients in HFS. Vein and artery were responsible TGN in 34 patients and 14 patients (3.8%) in HFS. Unspecified small arteries and veins only were responsible TGN in 3 patients and 4 patients in HFS.

In the TFF MVDS, out of the 430 patients who underwent the operations (Table 1), SCA was responsible for TGN in 310 patients and 3 patients HFS. AICA was responsible for TGN in 15 patients and 63 patients in HFS. PICA was responsible for TGN in 1 patient and 2 patients in HFS. LA was responsible TGN in 2 patients and 1 patient in HFS.

Additionally, out of the 430 patients in TFF MVDS, unspecified small arteries were responsible TGN in 4 patients and 2 patients in HFS. Vein only was responsible TGN in 5 patients and 19 patients in HFS. Vein and artery were responsible TGN in 30 patients and 10 patients in HFS. Unspecified small arteries and veins only were responsible TGN in 4 patients and 2 patients in HFS.

### 3.4. Follow-ups, complications and recurrences

**Figure 2.**
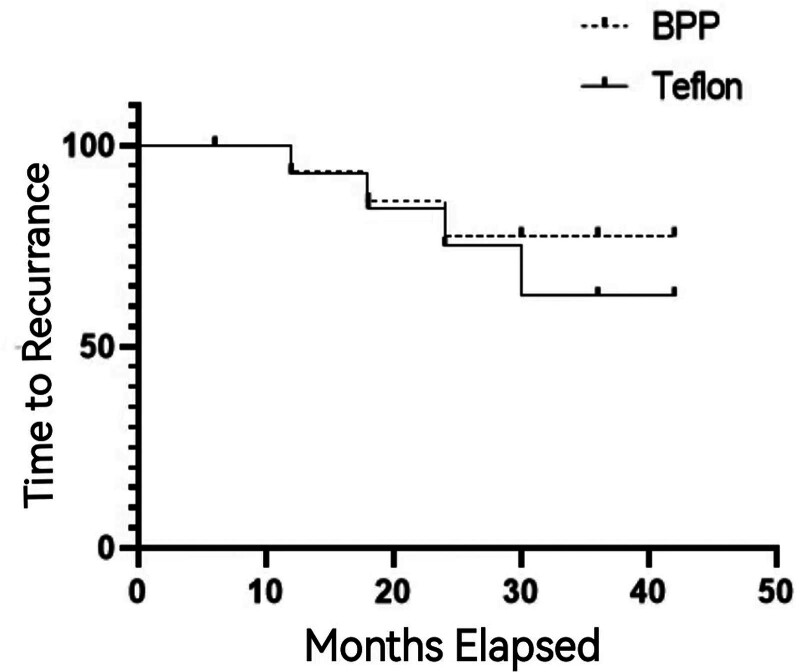
The time of recurrence (TOR) was almost the same in both groups until after 30 mo onwards where difference was observed.

**Figure 3.**
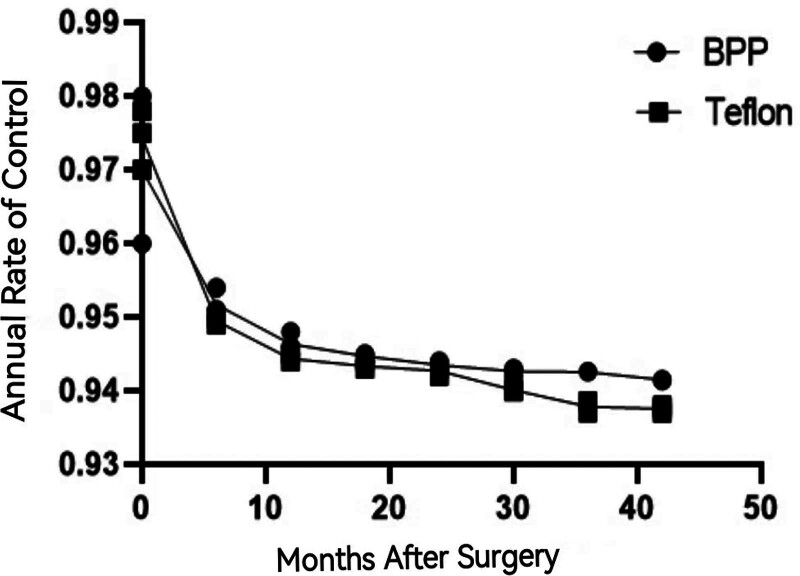
Recurrence of MVD; the annual rate of control (AROC) of symptomatology was almost the same in both groups until after 30 mo onwards where difference was observed. MVD = microvascular decompression.

All the patients in both the BPP MVDS and the TFF MVDS were followed for 42 months (Figs. 2 and 3). No patients were lost on follow-up. We recorded no mortality in the patients who underwent BPP MVDS but only one mortality in the TFF MVDS. The cause of death was not related to the surgery. In both groups, one patient each developed cerebellar hematoma. The hematomas did not trigger any significate symptomatology necessitating reoperations. Also, one patient in the BPP MVDS developed cerebellar edema while 2 patients in the in the TFF MVDS also developed cerebellar edema (Table 2). The cerebellar edemas did not trigger any significate symptomatology necessitating surgical intervention and were managed conservatively.

**Table 2:** Complications and recurrence of all the MVDS.

Parameters	BPP MVD (N = 368)	TFF MVD (N = 430)	*P* value
Death	0	1	.4896
Brain-stem infarct	0	0	.5996
Cerebellar hematoma	1	1	.5698
Cerebellar edema	1	2	.3427
Hydrocephalus	0	0	.5996
Facial paresis	2	2	.4796
Hearing loss	3	2	.3597
CSF leak	8	6	.4246
Intracranial infection	4	3	.4685
Implant displacement	0	3	.2578
Recurrence and granuloma formation	0	18	.0135

There are no significate statistical differences in mortality, brain stem infarction, cerebellar edema, hydrocephalus, facial paresis, hearing loss, CSF leakage, infection as well as implant displacement between the BPP MVD and the TFF MVD. However, there is significate statistical differences in recurrence rate and granuloma formation in the 2 groups. Statistical analysis was done as using the Mann–Whitney test.

BPP = bovine pericardial patch, CSF = cerebrospinal fluid, MVDS = microvascular decompression surgery, TFF = Teflen felt.

Additionally, 2 patients in the BPP MVDS developed facial paresis while 2 patients in the in the TFF MVDS also developed facial paresis. Also, 3 patients in the BPP MVDS developed hearing loss while 2 patients in the in the TFF MVDS also developed hearing loss. There was no statistically significant difference in the occurrence of facial paresis in both groups (*P* = .4796). Moreover, 8 patients in the BPP MVDS had CSF leakage while 6 patients in the TFF MVDS also had CSF leakage (*P* = .4246). Furthermore, 4 patients in the BPP MVDS developed intracranial infections while 3 patients in the in the TFF MVDS also developed intracranial infections (*P* = .4685) as shown in Table 2. All the patients who developed intracranial infections did not require reoperation and were treated conservatively.

No patients developed brain-stem infarction and hydrocephalus in both groups. Interesting, while no patient developed granuloma formation in the BPP MVDS, while 18 patients developed granuloma formation in the TFF MVDS and this was statistically significant (*P* = .0135) as indicated in Table 2. Notably, we did not observe recurrence in the BPP MVDS. However, 18 cases of recurrences were observed in the TFF MVDS (*P* = .0135). Interestingly, all the recurrent cases also developed granulomas. Thus, the granulomas triggered the recurrences in all the cases that recurred. All cases with recurrences were successfully reoperated and they are well now.

Furthermore, we did not observe implant displacement in the BPP MVDS, however, we observed 3 in the TFF MVDS. Also implant displacement correlated with recurrence of symptoms in the 3 patients. The time of recurrence (TOR) was almost the same in both groups until after 30 months onwards where difference was observed (Fig. 2). Interestingly, almost all recurrences also occurred at this same time point. Also, the annual rate of control (AROC) of symptomatology was almost same in the in the TFF MVDS compared to the BPP in eggroll fashion group until 30 months onwards where difference was observed. Notably, almost all the recurrences also occurred at this same time point (Fig. 3). The TFF MVDS had significantly higher recurrence and granuloma rates (4.2% vs 0%; *P* = .0135, 95% CI: 1.8–6.5%). Adjusted hazard ratio (aHR) accounting for follow-up duration: 0.12 (95% CI: 0.03–0.51).

Notably, the complication rate in the BPP MVDS was 19/368 which constituted 5.16% while the complication rate in the TFF MVDS was 38/430 which constituted 8.84%. The overall complication rate was 57/798 which constituted 7.14%. We did not observe significant difference in LOS between the BPP MVDS and the TFF MVDS (*P* = .146) as shown in Table 3. Interestingly, we observed significant difference in the post-surgical cost (*P* = .0356) and total cost (*P* = .00135) between the BPP MVDS and the TFF MVDS as shown in Table 3. Post-surgical cost and total hospital cost was higher in the TFF MVDS because of the relatively high complication rate in the in the TFF MVDS. BPP MVDS showed a ‌88% lower recurrence risk vs. TFF MVDS (HR = 0.12, *P* = .0135) and veins showed a non-significant trend toward higher risk versus arteries (HR = 1.45, *P* = .15) as shown in Table 4.

**Table 3:** Show the length of hospital stay in days and cost (RMB) comparison between the BPP MVD and TFF MVD.

Parameters	BPP MVD [N = 368] Mean (SD)	TFF MVD [N = 430] mean (SD)	Difference in mean	*P* value
Length of hospital stay (d)	7 (5)	8 (6)	2	.146
Total cost (RMB)	56,038 (16,306)	77,608 (30,620)	21,570	.00135*
Pre-hospital (RMB)	298 (180)	482 (997)	92	.234
Operation (RMB)	19,526 (2870)	19,364 (4061)	−82	.283
Post-surgery (RMB)	36,214 (2044)	57,762 (3519)	972	.0356*

Costs were adjusted to 2023 RMB using China’s consumer price index (cumulative inflation 2015–2022: 15.2%).

BPP = bovine pericardial patch, CI = confidence interval, HR = hazard ratio, MVD = microvascular decompression, SD = standard deviation, TFF = Teflen felt.

* *P* < .05.

**Table 4:** Multivariable Cox regression analysis adjusted for specified covariates.

Variable	Adjusted HR (95% CI)	*P*-value
Intervention		
BPP vs TFF	0.12 (0.03–0.51)	.0135
Vessel type		
Vein vs Artery	1.45 (0.87–2.41)	.15
Age (per 1-yr increase)	1.02 (0.98–1.06)	.28
Sex (male vs female)	0.89 (0.54–1.47)	.64
Symptom duration (mo)	1.03 (0.99–1.07)	.11

BPP MVD showed a 88% lower recurrence risk vs TFF (HR = 0.12, *P* = .0135). Veins showed a non-significant trend toward higher risk vs arteries (HR = 1.45, *P* = .15).

BPP = bovine pericardial patch, CI = confidence interval, HR = hazard ratio, MVD = microvascular decompression, TFF = Teflen felt.

## 4. Discussion

The TFF MVDS had significantly higher recurrence and granuloma rates compared to the BPP MVDS. Post-surgical cost and total hospital cost was higher in the TFF MVDS compared to the BPP MVDS because of the relatively high complication rate in the in the TFF MVDS. Also, the BPP MVDS was not associated with implant displacement compared to the TFF MVDS. Furthermore, no mortality was recorded in the BPP MVDS compared to one mortality in the TFF MVDS. However, the cause of death was not related to the surgery. Thus, BPP roll around the nerve like an eggroll and fixed with a closing suture could be a good alternative for TFF dependent MVDS.

MVDS is a microsurgical technique that involves the insertion of small prostheses between the blood vessels and the cranial nerves or the brainstem to relieve patients of disabling facial pain as well as spasm. Hasegawa et al revealed that nonabsorbable materials such as polytetrafluoroethylene (TFF), polyurethane, expanded polytetrafluoroethylene, and silk thread as well as absorbable material such fibrin glue, cellulose, gelatin, and collagen are the widely used prosthesis in Japan.^[2]^ Also, it worth noting that TFF is the most commonly used prosthesis for MVDS all over the world.^[6,24–26]^ However, BPP has widely been used for dural graft^[20–22]^ and vascular reconstructions.^[23]^

These prostheses above are occasionally associated with insufficient decompression, granuloma formation around the implants, as well as adhesive inflammation and arachnoiditis which triggers recurrence necessitating a second operation is most cases.^[1,8–19]^ Also, a case of degeneration of polyurethane, used in MVDS which resolved in delayed recurrence of HFS has been reported.^[27]^ Thus, we postulated that, a BPP roll around the nerve like an eggroll and fixed with a closing suture may solve the migration problem and provide a good alternative for TFF dependent MVDS because of its less tendency to migrate.

Interestingly, were observed significant differences in recurrence rate and granuloma formation between the 2 groups. Notably, the same cases that developed granuloma also recurred in the TFF MVDS. Thus, the granulomas triggered the recurrences in all the cases that recurred. The TOR and the AROC of symptomatology were almost the same in both groups until after 30 months onwards where difference was observed. Thus, the draw backs associated with TFF MVDS such as insufficient decompression, granuloma formation around the implants, which triggers adhesive inflammation and arachnoiditis leading to recurrence^[1,8–19]^ were not seen in the BPP in eggroll fashion MVDS. In the TFF MVDS, TFM plates were used to prevent the TFF from having direct contact with the nerve.

Oiwa et al who initially performed MVDS using a dural substitute did not detect recurrences in their patients during a follow-up period of 13-70 months with a mean follow-up period of 42.3 months, signifying that the dural substitute continues to exert its shock-absorbing effect for a long time with little inflammatory reaction or granuloma formation that is considered to cause recurrence.^[1]^ This technique is easier than sling retraction in the MVDS and TFF prosthesis because it does not necessitate that the vessels be moved away from the cranial nerves.

The prosthesis is not firm or rigid and so it is not harmful to the cranial nerves most especially the trigeminal nerve. Furthermore, the prosthesis can be inserted in between the nerve and the vessel involved even if the compression sites are situated in the mid-third of the trigeminal nerve, as often experienced in TGN. Nevertheless, temporary facial hypesthesia usually occurs in a few patients in whom the prosthesis enclosed the nerve directly.^[1,28]^ We observed that, 0.54% of our patients in the BPP MVDS developed facial paresis while 0.46% of the patients in the TFF MVDS also developed facial paresis.

However, there was no statistically significant difference in the occurrence of facial paresis between the BPP MVDS and TFF MVDS (*P* = .4796). It is worth noting that the normal BPP is 5cm × 5cm which is more than enough to for dura repair thus, we utilized same BPP for nerve insulation and dura repairs in our patients with no additional cost to the patients. Furthermore, we did not observe migrations of the BPP because we fixed the BPP’s with closing suture after wrapping it loosely around the nerve. Intraoperative EMG as well ABRs for monitoring the cranial nerves is very crucial in all MVDS.^[29]^

Interestingly, SCA, AICA, PICA, LA, unspecified small arteries, vein only, vein and artery as well as unspecified small arteries and veins were responsible for both TGN and HFS as well as in both the BPP group and the TFF group. It is well established that, patients with venous compression may have relatively less pain improvement and higher recurrence rates compared to those with arterial compression.^[30,31]^ However, we did not observe any significate difference in responsive vessels in the TGN and HFS as well as the BPP MVDS and the TFF MVDS.

Comparatively, in both TGN and HFS patients as well as in both the BPP MVDS and the TFF MVDS respectively, sex, preoperative duration of symptoms, age at onset of symptoms, age at surgery as well as the side of operation were not statistically significant. Also, 98.87% of our patients agreed to PDT before surgeries while 1.13% denied any PDT before surgeries in both TGN and HFS patients. The TOR and the AROC of symptomatology were almost same in both groups until after 30 months onwards where difference was observed. Interestingly, recurrences also occurred at this same time point.

It is advisably that in all patients undergoing MVDS, the preoperative screening should include MRI or CT scan to exclude a structural pathology like meningiomas, acoustic neuromas, or an epidermoid tumor which may also cause facial nerve irritation.^[5,29]^ Also, the consideration of posterior fossa exploration is often appropriate even if a high-resolution MRI is unable detect a causative vascular loop.^[5]^ All our patients underwent brain both MRI as well as CT and we did not identify any structural pathologies prior to surgeries.

They were no significant statistical differences in mortality, brain stem infarction, cerebellar edema, hydrocephalus, facial paresis, hearing loss, CSF leakage, infection as well as implant displacement between the BPP MVDS and the TFF MVDS. However, there is significant statistical differences in recurrence and granuloma formation between the 2 groups. The major complications associated with MVDS as reported includes cerebellar injury, hearing loss, and CSF leakage.^[32]^ Other rare complications associated with MVDS includes, facial weakness or anesthesia as well as lower cranial nerve dysfunction.^[18,19,33]^ All cases with recurrences were successfully reoperated and they are well now.

Notably, the complication rate in the BPP MVDS was relatively lower than the complication rate in the TFF MVDS. However, the overall complication rate was 7.14%.

We did not observe significant difference in LOS between the BPP MVDS and the TFF MVDS. Notably, we observed significant difference in the post-surgical cost and total cost between the BPP MVDS and the TFF MVDS. Post-surgical cost and total hospital cost was higher in the TFF MVDS because of the relatively high complication rate in the in the TFF MVDS.

This study has limitations: retrospective design may introduce selection bias; outcomes were assessed by unblinded clinicians; long-term efficacy (>5 years) remains unknown. A multicenter prospective study with a larger sample size will be more conclusive. However, Oiwa et al who initially performed MVDS using a dural substitute indicates the procedure is safe and beneficial to patients.^[1]^ BPP’s cost-effectiveness in China may not translate directly to other regions (e.g., TFF MVDS costs $300 to 500 USD in Western countries vs ¥2000 RMB here). Surgical training in the “eggroll” technique is also critical for adoption.

## 5. Conclusion

BPP roll around the nerve like an eggroll and fixed with a closing suture could be a good alternative for TFF dependent MVDS. BPP MVDS demonstrates superior clinical outcomes with lower recurrence and costs compared to TFF MVDS. Further studies should investigate its biomechanical properties and long-term efficacy. The absence of granulomas in the BPP MVDS suggests reduced inflammatory responses compared to TFF MVDS.

## Author contributions

**Conceptualization:** Min Tang, Seidu A. Richard, Rong Liu, Zhigang Lan, Heng Zhang, Ding Lei.

**Data curation:** Min Tang, Seidu A. Richard, Rong Liu, Zhigang Lan, Heng Zhang, Ding Lei.

**Formal analysis:** Min Tang, Seidu A. Richard, Zhigang Lan, Heng Zhang.

**Funding acquisition:** Zhigang Lan.

**Investigation:** Min Tang, Seidu A. Richard, Rong Liu, Heng Zhang, Ding Lei.

**Methodology:** Seidu A. Richard, Rong Liu, Zhigang Lan, Heng Zhang, Ding Lei.

**Resources:** Heng Zhang.

**Software:** Zhigang Lan.

**Supervision:** Ding Lei.

**Writing – original draft:** Seidu A. Richard.

**Writing – review & editing:** Min Tang, Seidu A. Richard, Rong Liu, Zhigang Lan, Heng Zhang, Ding Lei.
